# Targeting MCT4 to reduce lactic acid secretion and glycolysis for treatment of neuroendocrine prostate cancer

**DOI:** 10.1002/cam4.1587

**Published:** 2018-06-14

**Authors:** Stephen Yiu Chuen Choi, Susan L. Ettinger, Dong Lin, Hui Xue, Xinpei Ci, Noushin Nabavi, Robert H. Bell, Fan Mo, Peter W. Gout, Neil E. Fleshner, Martin E. Gleave, Colin C. Collins, Yuzhuo Wang

**Affiliations:** ^1^ The Vancouver Prostate Centre Vancouver General Hospital The University of British Columbia Vancouver BC Canada; ^2^ Department of Urologic Sciences The University of British Columbia Vancouver BC Canada; ^3^ Department of Experimental Therapeutics BC Cancer Research Centre Vancouver BC Canada; ^4^ Division of Urology Department of Surgical Oncology Princess Margaret Cancer Centre University Health Network and the University of Toronto Toronto ON Canada

**Keywords:** cancer‐generated lactic acid, MCT4, neuroendocrine prostate cancer, patient‐derived xenografts, reprogrammed cancer metabolism

## Abstract

Development of neuroendocrine prostate cancer (NEPC) is emerging as a major problem in clinical management of advanced prostate cancer (PCa). As increasingly potent androgen receptor (AR)‐targeting antiandrogens are more widely used, PCa transdifferentiation into AR‐independent NEPC as a mechanism of treatment resistance becomes more common and precarious, since NEPC is a lethal PCa subtype urgently requiring effective therapy. Reprogrammed glucose metabolism of cancers, that is elevated aerobic glycolysis involving increased lactic acid production/secretion, plays a key role in multiple cancer‐promoting processes and has been implicated in therapeutics development. Here, we examined NEPC glucose metabolism using our unique panel of patient‐derived xenograft PCa models and patient tumors. By calculating metabolic pathway scores using gene expression data, we found that elevated glycolysis coupled to increased lactic acid production/secretion is an important metabolic feature of NEPC. Specific inhibition of expression of MCT4 (a plasma membrane lactic acid transporter) by antisense oligonucleotides led to reduced lactic acid secretion as well as reduced glucose metabolism and NEPC cell proliferation. Taken together, our results indicate that elevated glycolysis coupled to excessive MCT4‐mediated lactic acid secretion is clinically relevant and functionally important to NEPC. Inhibition of MCT4 expression appears to be a promising therapeutic strategy for NEPC.

## 1 INTRODUCTION

Reprogrammed energy metabolism has long been associated with cancer and is considered an important hallmark.^1^ Elevated glucose consumption coupled to increased lactic acid production (aerobic glycolysis) is thought to facilitate incorporation of nutrients into the biomass of cancer cells in order to produce new cells.^2^ However, excessive lactic acid secretion can also stimulate multiple cancer‐promoting processes, including local tissue invasion, metastasis, neoangiogenesis, resistance to hypoxia, and altered epigenetics.^3^, ^4^ Furthermore, as previously suggested, acidification of the cancer cell environment by increased cancer‐generated lactic acid secretion can suppress the local anticancer immune response.^3^ Given these wide‐ranging downstream effects critical to cancer biology, inhibition of lactic acid secretion offers a promising therapeutic strategy potentially applicable to multiple cancer types.

Prostate cancer (PCa) is the most commonly diagnosed noncutaneous cancer and a leading cause of cancer death for North American men.^5^ While the majority of advanced PCas are initially androgen ablation‐sensitive, many become castration‐resistant prostate cancers (CRPCs). Recent use of increasingly potent antiandrogen receptor therapeutics has further promoted transdifferentiation into androgen‐independent neuroendocrine prostate cancers (NEPCs) as a mechanism of treatment resistance,^6^ potentially accounting for 16%‐25% of future CRPCs.^7^ Unfortunately, NEPC is a highly aggressive disease currently lacking effective treatment, with a median patient survival of <1 year.^7^ As such, the emergence of NEPC constitutes a major problem in the clinical management of advanced PCa.

The current, limited understanding of NEPC biology, and in particular NEPC energy metabolism, is a key hurdle in developing effective therapy for this advanced PCa subtype. However, we recently found that increased secretion of lactic acid, as facilitated by elevated expression of the plasma membrane transporter monocarboxylate transporter 4 (MCT4), was relevant to the development of CRPC, offering MCT4 expression as a promising target for therapeutic intervention.^8^ Following up this discovery, we investigated whether elevated glycolysis and increased MCT4‐mediated lactic acid secretion are clinically relevant to NEPC and amenable to inhibition as its treatment strategy.

## 2 MATERIALS AND METHODS

### 2.1 Patient‐derived xenograft PCa models and RNA microarray

Original patient PCa specimens were grafted under subrenal capsules of male NOD/SCID mice and serially transplanted as described.^9^ Detailed characteristics of these patient‐derived xenograft (PDX) PCa models can be found on the Living Tumour Laboratory website (http://livingtumorcentre.com/index.html). RNA microarray analyses of NEPC and PCa adenocarcinoma PDXs were done using Agilent Sure‐Print G3 Human GE 8x60K Microarray Design ID 028004 following previous protocols.^9^

### 2.2 Metabolic pathway scores

Calculation of metabolic pathway scores has been previously described in detail.^10^ For comparing alterations after neuroendocrine (NE)‐transdifferentiation in the LTL‐331/331R model,^6^ the log2 fold‐changes of each gene in the respective metabolic pathways were averaged. For comparisons between NEPC and PCa adenocarcinoma PDXs, gene expression *z*‐scores of NEPC PDXs were derived from the Mean/SD of 8 adenocarcinoma PDXs. The *z*‐scores of each gene in the respective metabolic pathways were averaged to arrive at a pathway score for each NEPC PDX model. The average metabolic pathway scores for all NEPC PDX models were then taken to indicate an overall metabolic pathway change. For patient NEPC samples, a similar calculation was done using publically available gene expression data from Beltran et al,^11^ with gene expression *z*‐scores derived from the Mean/SD of 30 patient adenocarcinoma samples.

### 2.3 NCI‐H660 cell cultures

NCI‐H660 NEPC cells were grown in RPMI‐1640 (Hyclone) containing 5% fetal bovine serum (GIBCO), 1% Insulin‐Transferrin‐Selenium (Thermo Fisher), 10 nmol/L hydrocortisone (Sigma), 10 nmol/L beta‐estradiol (Sigma), and 1% Matrigel (Corning). Cells were dissociated for passaging and various assays using Accutase (Stemcell Technologies).

### 2.4 Transfection, proliferation, and metabolic assays

First‐generation antisense oligonucleotides (ASOs) were synthesized by Eurofin with fully phosphorothioated backbones. The sequences have been published.^8^ NCI‐H660 cells were transfected with ASO (0.5 nmol) using RNAiMAX in serum‐free media containing 2000 mg/L (11 mmol/L) of glucose. At 96 hours post–transfection, cells were dissociated and counted using the TC20 Automated Cell Counter (Bio‐Rad). Cell viability was assessed by trypan blue exclusion. Media samples were also collected 96 hours after transfection. Lactate and glucose concentrations were measured using corresponding colorimetric assay kits from Biovision.

### 2.5 Gene expression analysis by qPCR

NCI‐H660 cells were harvested 96 hours after ASO transfection and RNA was isolated using RNeasy Mini Kit (Qiagen). cDNA was synthesized from 1 μg of total RNA using the QuantiTect Reverse Transcription Kit (Qiagen). qRT‐PCR reactions using KAPA SYBR Fast Universal (Kapa Biosystems) were performed in triplicate in a ViiA 7 Real‐Time PCR system (Applied Biosystems).

## 3 RESULTS AND DISCUSSION

### 3.1 Glycolysis and lactic acid production pathways are upregulated in PDX NEPC models

**Figure 1:**
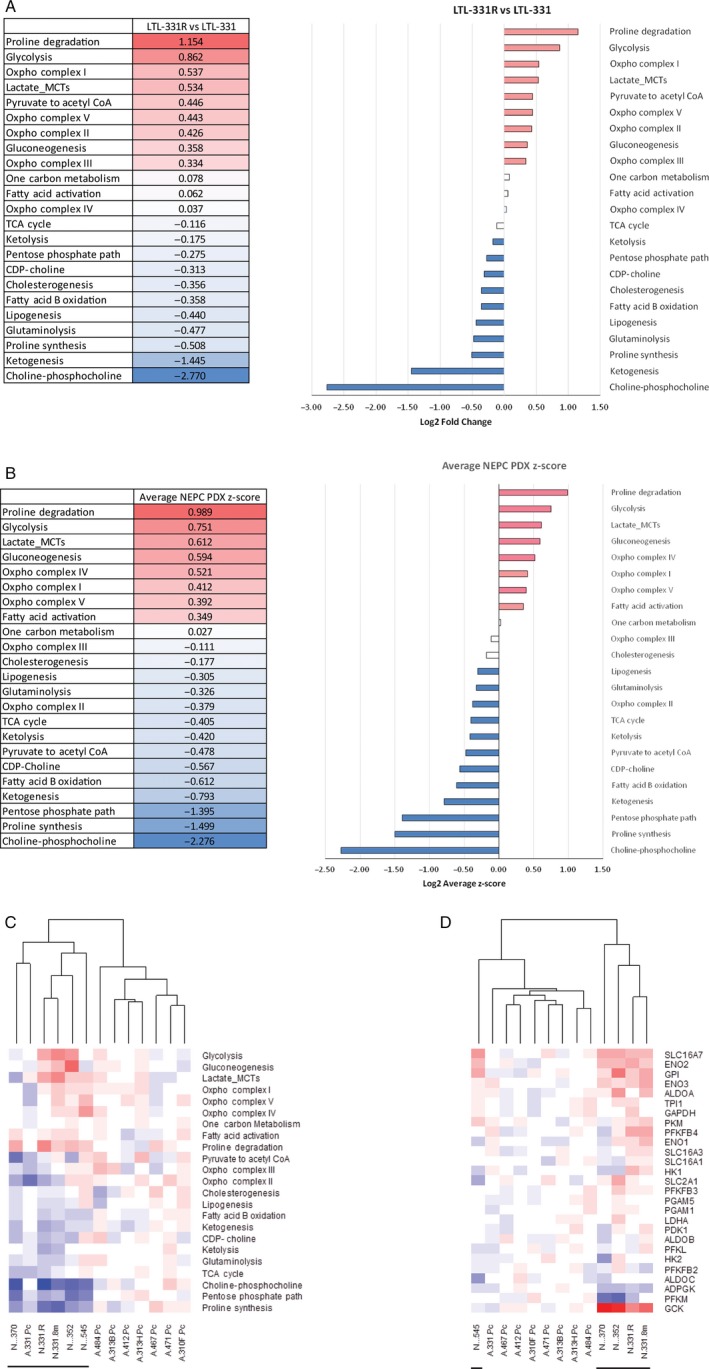
Glycolysis and lactic acid production pathways are among the upregulated metabolic pathways in patient‐derived xenograft (PDX) models of neuroendocrine prostate cancer (NEPC). A, A comparison of the transdifferentiated NEPC xenografts (LTL‐331R) with the parental prostate cancer (PCa) adenocarcinoma PDX (LTL‐331) was done by taking an average of the log2 fold‐change of each gene in the respective metabolic pathway. Glycolysis and lactic acid production, together with other components of glucose metabolism, are upregulated in NEPC. Conversely, ketogenesis and choline metabolism were the most downregulated metabolic pathways. B, An expanded analysis averaging the metabolic pathway scores of 5 NEPC PDX models as compared to 8 PCa adenocarcinoma PDX models revealed that glycolysis and lactic acid production remained among the upregulated metabolic pathways, suggesting that this phenomenon is consistent across different NEPC PDXs. C, A hierarchical clustering analysis of the different PDX models using metabolic pathway scores separated NEPC PDXs (underlined) from PCa adenocarcinoma PDXs, suggesting that NEPC metabolism is indeed distinct from PCa adenocarcinoma. Interestingly, the parental LTL‐331 PDX model clustered together with the NEPC PDXs, indicating that it may be metabolically predisposed toward NEPC transdifferentiation. D, A hierarchical clustering analysis using only genes from the glycolysis and lactic acid production pathways distinguished NEPC PDXs from adenocarcinoma PDXs, indicating that elevated glycolysis may be an important metabolic phenotype of NEPC

We previously developed the first‐in‐field LTL‐331/331R PDX NEPC transdifferentiation model. After castration of mice bearing hormone‐naive, androgen‐ablation‐sensitive LTL‐331 prostatic adenocarcinoma, the malignancy spontaneously recurs as a typical androgen‐independent NEPC (LTL‐331R).^6^ Changes to metabolic pathway scores following NEPC transdifferentiation revealed that glucose‐related pathways, including glycolysis, lactic acid production, and components in oxidative phosphorylation comprise the majority (8 of 9, 89%) of upregulated metabolic pathways in LTL‐331R (Figure 1A). When the analysis was expanded to 5 NEPC PDXs and 8 PCa adenocarcinoma PDXs, the collective NEPC metabolic pathway scores indicated changes similar to those observed in LTL‐331R, with the majority (6 of 8, 75%) of upregulated metabolic pathways in NEPC PDXs remaining glucose‐related (Figure 1B). A hierarchical clustering analysis distinguished NEPC PDXs from PCa adenocarcinoma PDXs, suggesting that NEPC metabolism is distinct from that of PCa adenocarcinoma (Figure 1C). Interestingly, a similar separation can be observed when using only genes in the glycolysis and lactic acid production pathways (Figure 1D), indicating that elevated aerobic glycolysis is significant to this unique NEPC‐associated metabolic profile.

### 3.2 Elevated glycolysis and lactic acid production are the top 2 upregulated pathways in clinical NEPC tumors

**Figure 2:**
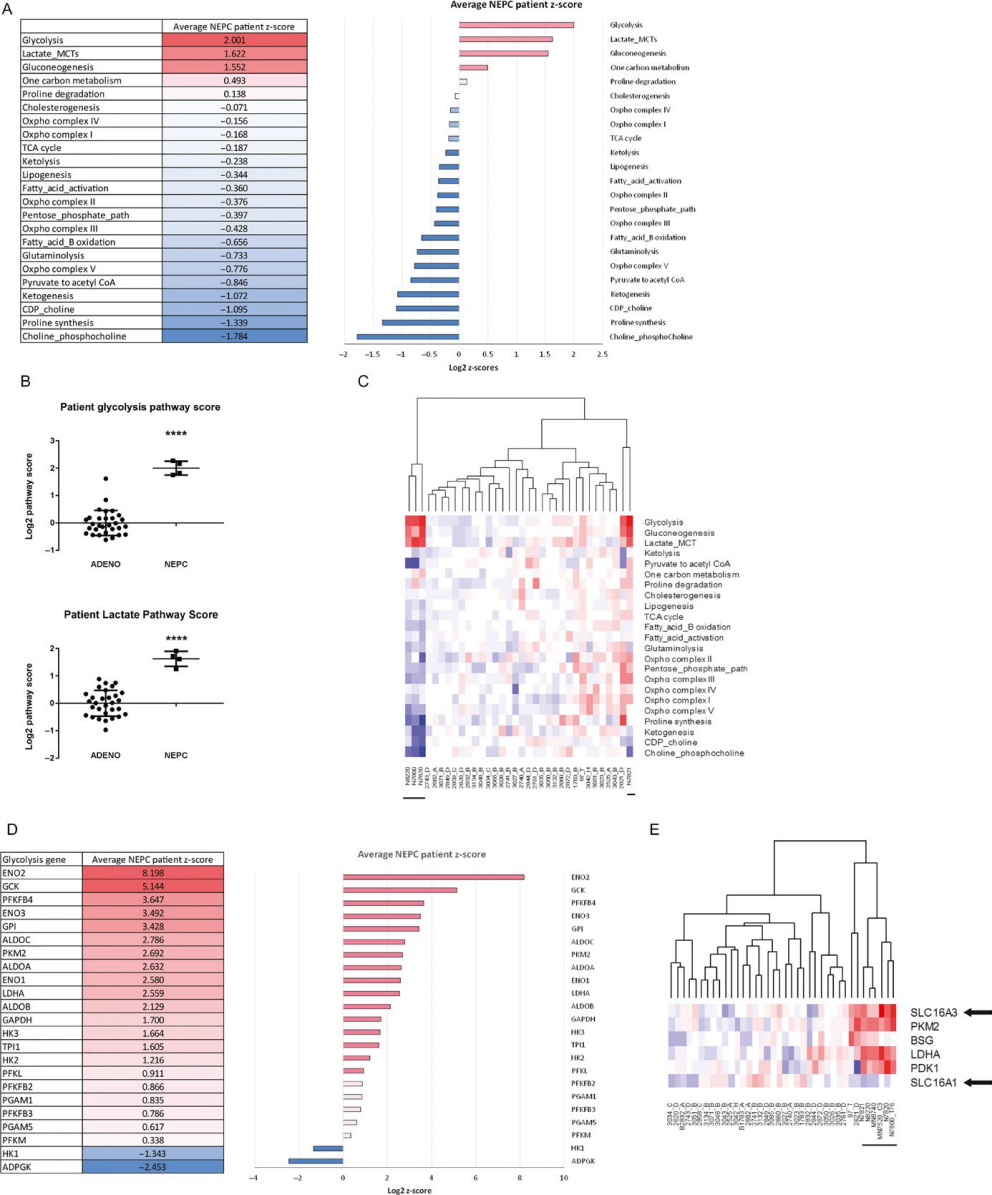
Elevated glycolysis and lactic acid production are even more prominent in patient neuroendocrine prostate cancer (NEPC) tumors and seem to be facilitated by overexpression of MCT4. A, A similar analysis calculating metabolic pathway scores using published patient NEPC gene expression data revealed that glycolysis and lactic acid production are the 2 most highly upregulated metabolic pathways in patient NEPC tumors. B, Statistical analysis looking specifically at the glycolysis and lactic acid production pathways also showed that the pathway upregulation in NEPC patient tumors is statistically significantly compared to patient prostate cancer (PCa) adenocarcinoma; ****, *P* < .0001. C, A hierarchical clustering analysis using the metabolic pathway scores distinguished NEPC patient tumors from patient PCa adenocarcinoma tumors, suggesting that NEPC metabolism is indeed clinically distinct. D, A ranking of the various genes in the glycolysis pathway showed that most genes were upregulated and contributed to the elevated pathway score. E, A hierarchical clustering analysis using only genes in the lactic acid production pathway distinguished patient tumors containing NEPC (underlined) from PCa adenocarcinoma tumors and benign samples (B). Interestingly, of the 2 major lactic acid transporters, MCT4 (SLC16A3) was upregulated but MCT1 (SLC16A1) was downregulated in all NEPC tumors, suggesting that elevated glycolysis and lactic acid production is likely mediated through elevated expression of MCT4

To validate our findings, we performed the same metabolic pathway analysis using publically available gene expression data of patient NEPC and PCa adenocarcinomas.^11^ Strikingly, the only upregulated metabolic pathways in patient NEPC tumors were directly related to glucose utilization, with glycolysis and lactic acid production being the top 2 statistically significant upregulated metabolic pathways (Figure 2A,B). A hierarchical clustering analysis using metabolic pathway scores was also able to distinguish NEPC patient tumors from patient PCa adenocarcinomas similar to findings from our PDX models (Figure 2C). This further contributes to the growing body of experimental evidence that the LTL PDXs can closely mirror clinical and biological characteristics of patient tumors.^6^, ^9^, ^10^

A closer look at the glycolysis pathway in NEPC patient tumors revealed that the vast majority (21 of 23, 91%) of genes in both pathways are upregulated (Figure 2D). Importantly, using only genes in the lactic acid production pathway, an expanded clustering analysis was also able to distinguish samples containing NEPC from other tissue types (Figure 2E). This lends further evidence that elevated glycolysis and increased lactic acid production contribute to the distinct NEPC‐associated metabolic signature. Furthermore, this is in contrast to our previous finding that primary treatment‐naive PCa is heterogeneous in their metabolic profiles,^10^ with only some primary PCa PDXs and patient tumors exhibiting upregulated glycolysis and lactic acid production pathways. Interestingly, this upregulation of lactic acid production appears to be facilitated at least in part through elevated expression of MCT4 (SLC16A3), but not MCT1 (SLC16A1), as all NEPC patient samples showed increased MCT4 but decreased MCT1 expression (Figure 2E). As such, MCT4 may be a potential therapeutic target for inhibiting elevated aerobic glycolysis and could be used as an effective treatment strategy for NEPC.

### 3.3 Inhibition of MCT4 expression in NEPC cells reduces cell proliferation and inhibits glucose metabolism

**Figure 3:**
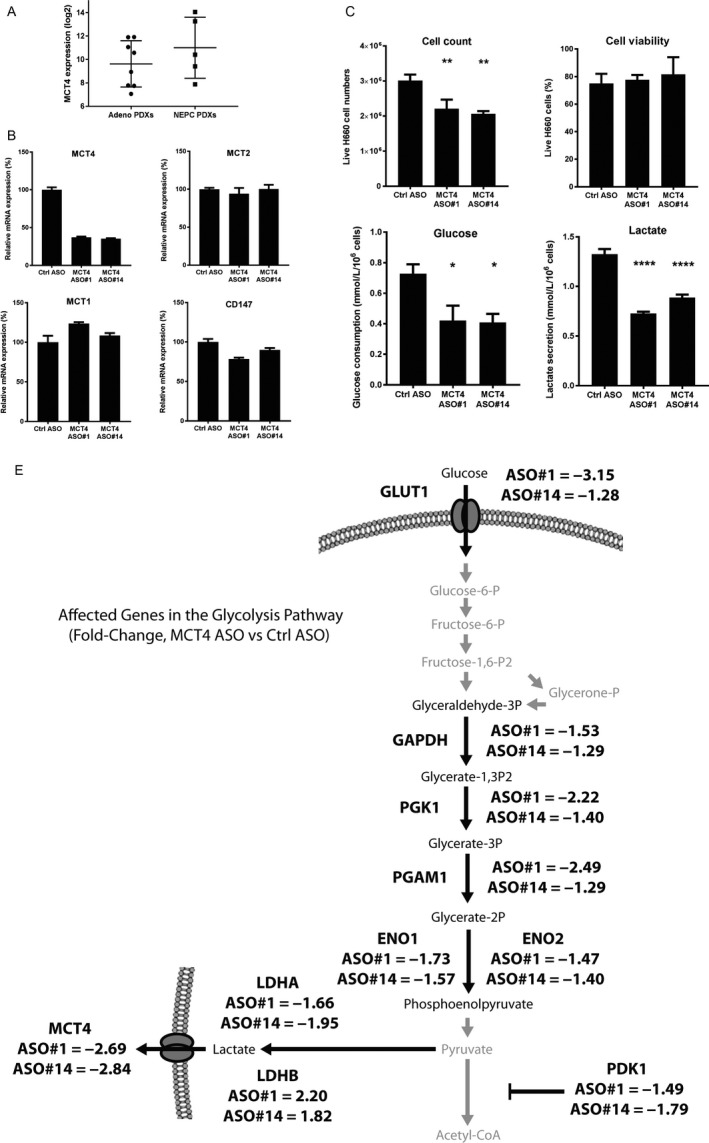
Inhibition of MCT4 expression in NCI‐H660 neuroendocrine prostate cancer (NEPC) cell lines reduced in vitro cell proliferation and inhibited glucose metabolism through downregulation of upstream genes in the glycolysis pathway. A, An increased average expression of MCT4 can be observed in the 5 NEPC PDXs when compared to the 8 adenocarcinoma PDXs used in this study. Line and error bars represent the mean and standard deviation, respectively. B, Transfection of MCT4 antisense oligonucleotides (ASOs) into H660 NEPC cells resulted in reduced expression of MCT4 after 96 h without affecting expression of other MCT family members or the accessory protein CD147. C, Transfection of MCT4 ASO resulted in the reduction of H660 cell proliferation measured 96 h post–transfection. Assessment of cell viability indicates no significant difference following treatment, suggesting a cytostatic effect. D, Inhibition of MCT4 expression also resulted in the reduction of glucose consumption and lactic acid secretion by H660 cells, indicating a disruption of glucose metabolism. E, A detailed analysis of the various genes in the glycolysis and lactic acid production pathways revealed that multiple upstream genes are affected following reduction of MCT4 expression. More specifically, reduced LDHA can contribute to reduced pyruvate conversion to lactate and increased LDHB expression can increase lactate conversion back to pyruvate. Additionally, reduced gene expression at various stages of glycolysis, including the glucose transporter GLUT1 and various glycolytic enzymes such as ENO1/2 and PGAM1 can also result in reduced consumption of glucose. Taken together, the inhibition of glycolysis and lactic acid production pathways appear to be a key mechanism of action following MCT4 inhibition. *, *P* < .05; **, *P* < .01; ****, *P* < .0001

We previously demonstrated that reduction of lactic acid secretion via MCT4 targeting is a promising therapeutic strategy for CRPC, resulting in inhibition of glucose metabolism, cell proliferation, and enhancement of anticancer immunity.^8^ We observed an increased MCT4 expression in the NEPC PDX models studied here. The LTL‐331R NEPC PDX tumor has a 2.56‐fold higher MCT4 expression compared to the parental LTL‐331 adenocarcinoma PDX tumor. Consistent with these observations, the average MCT4 expression in the 5 NEPC PDXs is also 2.61‐fold higher than the average expression in the 8 adenocarcinoma PDXs (Figure 3A). As such, we assessed the therapeutic efficacy of MCT4 targeting for treatment of NEPC using the NCI‐H660 NEPC cell line and our previously validated MCT4 ASOs.^8^ MCT4 ASO transfection was able to decrease MCT4 mRNA expression without affecting the levels of other MCT family members (Figure 3B). Inhibition of NEPC cell proliferation was also observed following MCT4 ASO treatment without inducing overt cytotoxicity, indicative of a cytostatic effect (Figure 3C). Characterization of the changes to glucose metabolism following MCT4 knockdown revealed a decrease in glucose consumption and lactic acid secretion (Figure 3D). A more detailed analysis of other genes in the glycolysis and lactic acid production pathways revealed that MCT4 inhibition resulted in downregulation of multiple upstream glycolytic genes. More specifically, reduced LDHA and increased LDHB expression can contribute to decreased pyruvate conversion to lactate while increasing lactate conversion back to pyruvate. Furthermore, reduced expression of PDK1 can also result in a metabolic switch away from lactic acid production toward increased glucose metabolism via the TCA cycle. Finally, reduced GLUT1 expression, together with reduced expression of other glycolytic enzymes, for example ENO1/2, PGAM1, and PGK1, can also contribute to decreased glucose utilization (Figure 3E). As such, MCT4 inhibition appears to inhibit NEPC cell proliferation in a cytostatic manner via inhibition of glucose metabolism—a similar mechanism as previously reported in CRPC cells.^8^

In addition to facilitating altered glucose metabolism, the MCT4 transporter also has important functions in regulating stress response, particularly under hypoxic conditions. While a detailed investigation into hypoxia is beyond the scope of our current study, recent literature reports have indicated that upregulation of MCT4 expression in PCa cells can be driven by HIF1α activation.^12^ As such, inhibition of MCT transporter functions in hypoxic tumor microenvironments could further contribute to the reduction of tumor growth in aggressive cancers like NEPC. Additionally, altered proline metabolism in the form of increased degradation and reduced biosynthesis appears to be another prominent NEPC‐associated metabolic feature. Further investigations into how these changes facilitate NEPC development could be a promising area of research, especially given its contribution to metastasis and the hypoxic response.^13^, ^14^ It has also been suggested that in prostatic tumors containing a mixture of NE and adenocarcinoma cells, certain paracrine factors secreted by NE cells could facilitate the growth and aggressiveness of neighboring adenocarcinoma cells.^15^, ^16^ Whether the increased secretion of lactic acid from NE cells could function in an analogous paracrine fashion to enhance PCa aggressiveness would be an intriguing area for further study. Finally, inhibition of glucose metabolism via CD44 has also been reported to sensitize NEPC cells to platinum‐based chemotherapy.^17^ The inhibition of MCT4 could thus, in a similar manner, further enhance the efficacy of existing PCa therapies. The contributions of these mechanisms, together with the effects of MCT4 inhibition in NEPC models in vivo*,* are being actively investigated in our laboratory.

In conclusion, our results suggest that elevated glycolysis and increased lactic acid production/secretion form a clinically relevant and functionally important NEPC metabolic phenotype. Given the many lactic acid‐associated tumor‐promoting processes, inhibition of elevated glycolysis and excessive MCT4‐mediated lactic acid secretion could be a much‐needed, potentially effective therapeutic strategy for treatment of NEPC and other transdifferentiated PCas. Furthermore, elevated aerobic glycolysis is a widespread metabolic feature common to multiple cancers.^18^, ^19^, ^20^ As such, an effective therapeutic strategy inhibiting lactic acid generation and causing subsequent reduction of proliferation, metastasis, angiogenesis, and suppression of anticancer immunity could have broad clinical applications beyond advanced PCa.

## CONFLICTS OF INTEREST

A patent on the MCT4 antisense oligonucleotide sequences used in this study has been filed by the University of British Columbia with YW, CC, PG, and SYCC as inventors. All other authors declare no conflicts of interest.
