# Supplementary material for: Targeting MCT4 to reduce lactic acid secretion and glycolysis for treatment of neuroendocrine prostate cancer
Source: Cancer Med. 2018 Jun 14;7(7):3385–92. doi: 10.1002/cam4.1587 (PMC6051138; doi:10.1002/cam4.1587)

Applied Biosystems
GeneMapper Software 5

Sample Name	Panel	Marker	OS	BIN	PHR	LPH	SPU	AN	BD	CC	OVL	GQ
4660	Identifiler_v1	AMEL			NA					NA		

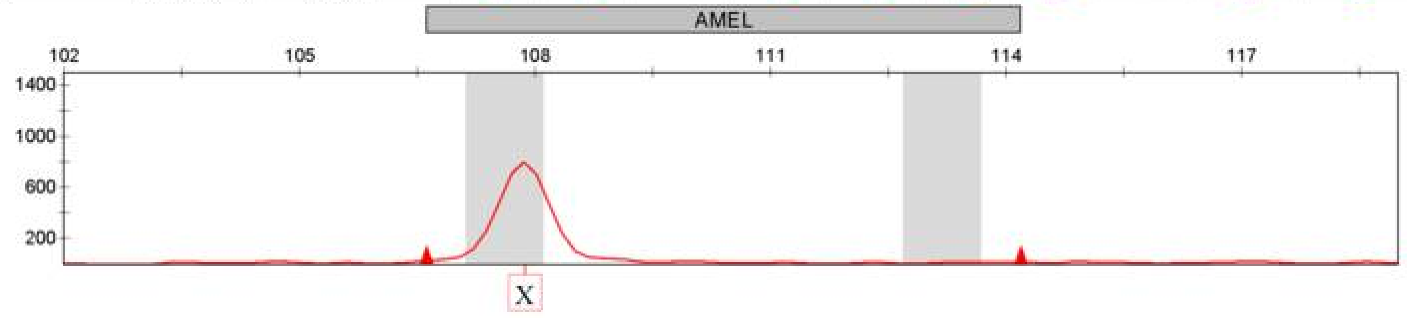

Sample Name	Panel	Marker	OS	BIN	PHR	LPH	SPU	AN	BD	CC	OVL	GQ
4660	Identifiler_v1	CSF1PO			NA					NA		

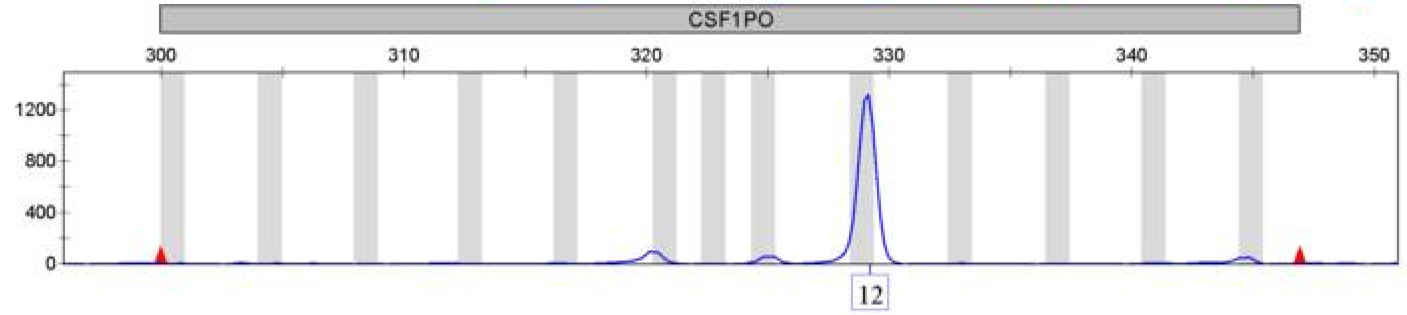

Sample Name	Panel	Marker	OS	BIN	PHR	LPH	SPU	AN	BD	CC	OVL	GQ
4660	Identifiler_v1	D13S317			NA					NA		

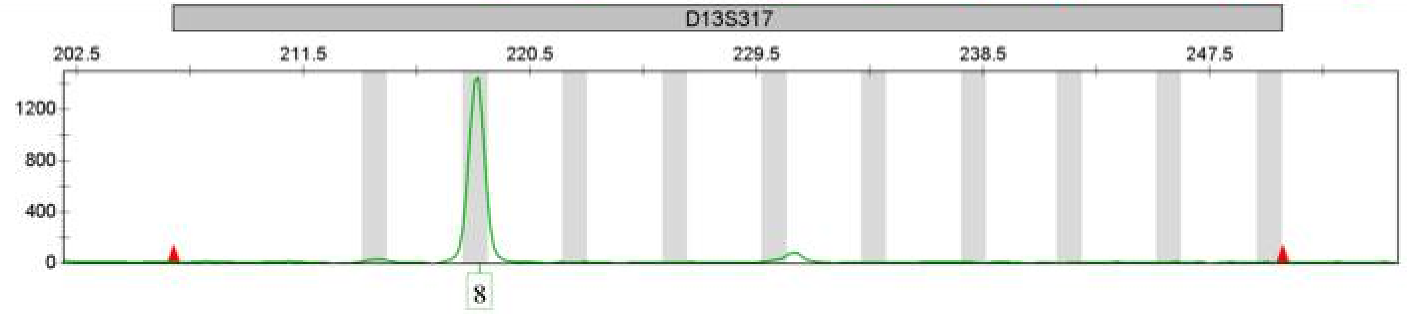

Sample Name	Panel	Marker	OS	BIN	PHR	LPH	SPU	AN	BD	CC	OVL	GQ
4660	Identifiler_v1	D16S539			NA					NA		

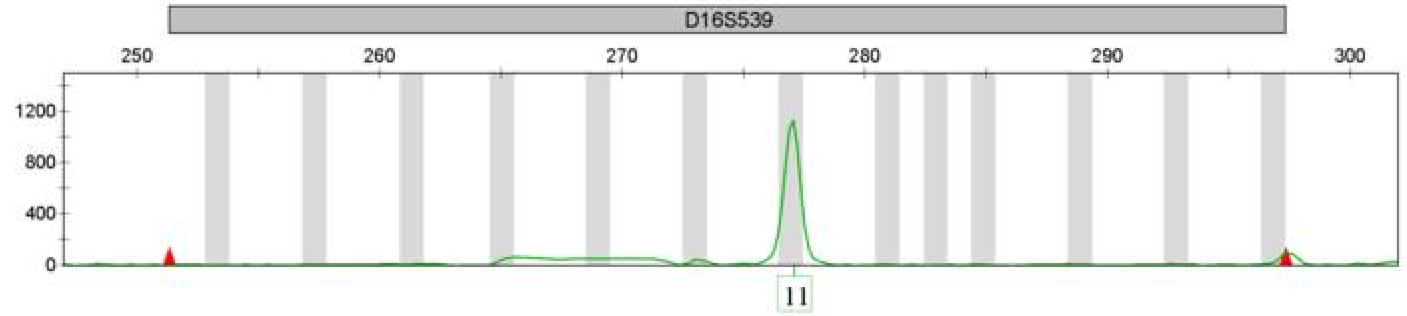

Sample Name	Panel	Marker	OS	BIN	PHR	LPH	SPU	AN	BD	CC	OVL	GQ
4660	Identifiler_v1	D19S433			NA					NA		

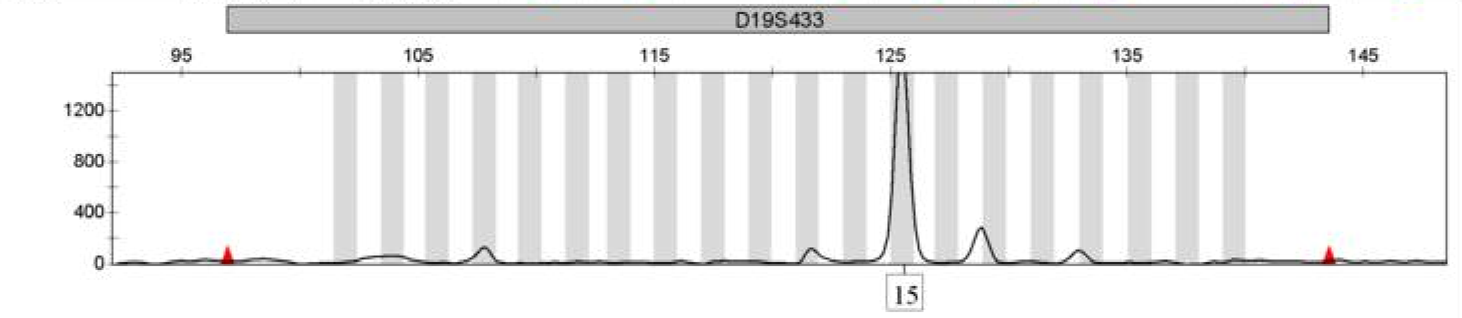

4660	Identifiler_v1	D3S1358								NA		

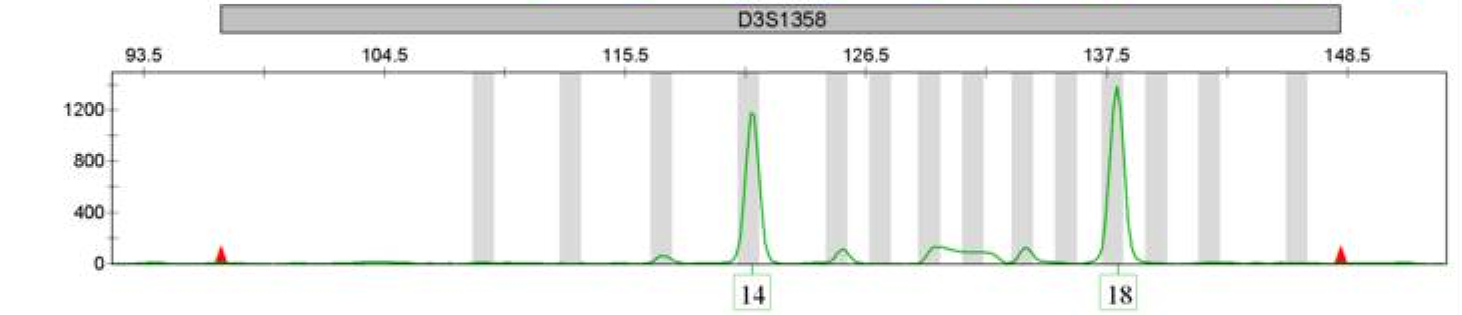

4660	Identifiler_v1	D7S820								NA		

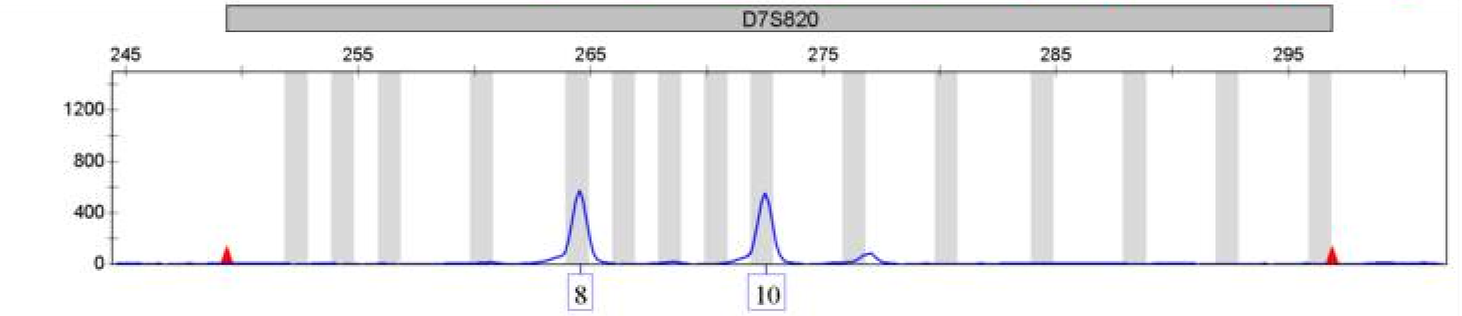

4660	Identifiler_v1	TPOX			NA					NA		

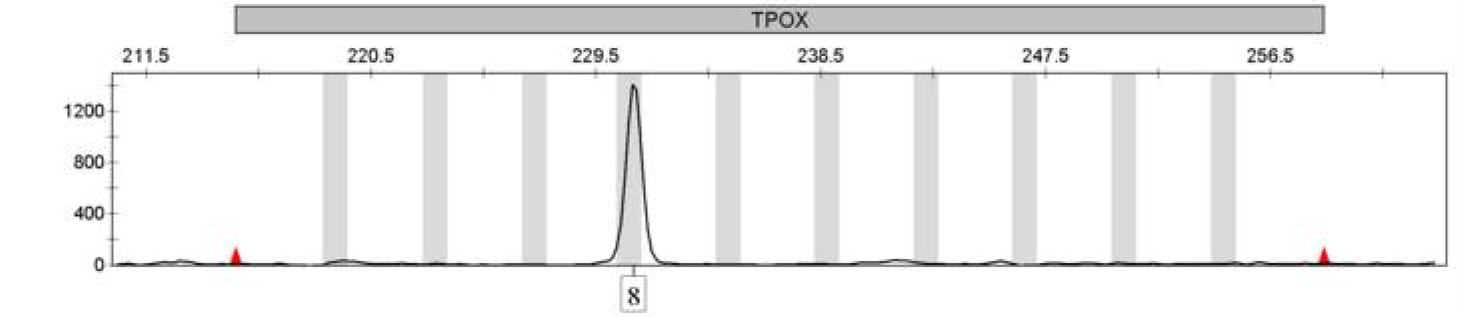

Sample Name	Panel	Marker	OS	BIN	PHR	LPH	SPU	AN	BD	CC	OVL	GQ
4660	Identifiler_v1	vWA			NA					NA		

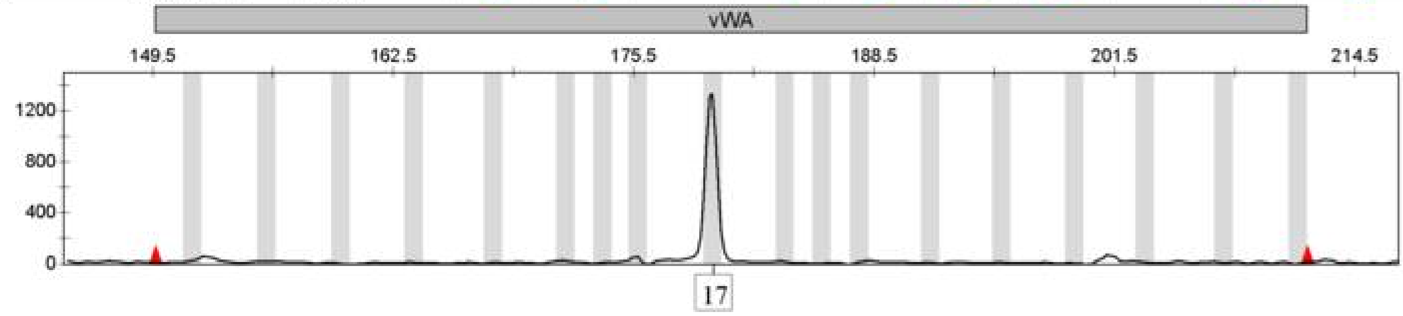

Sample Name	Panel	Marker	OS	BIN	PHR	LPH	SPU	AN	BD	CC	OVL	GQ
4660	Identifiler_v1	D18S51	-4.0	-4.0		-4.0	-4.0	-4.0	-4.0	NA		

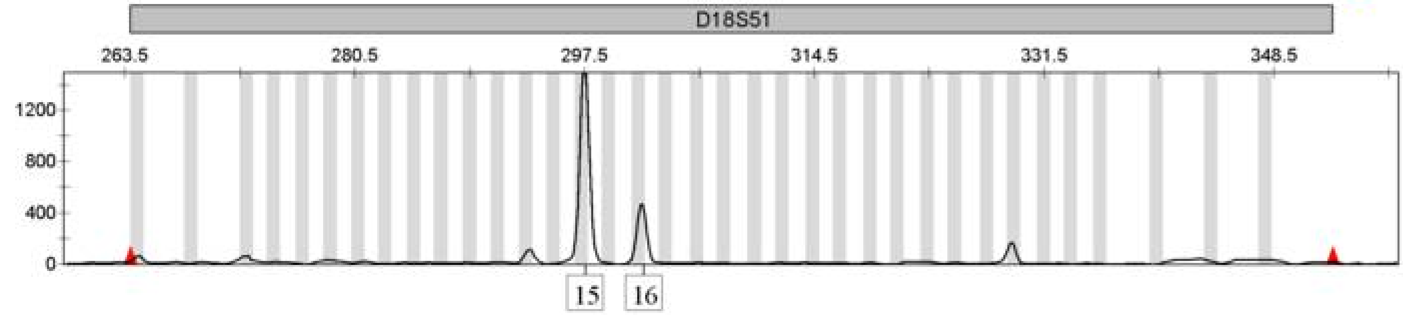

Sample Name	Panel	Marker	OS	BIN	PHR	LPH	SPU	AN	BD	CC	OVL	GQ
4660	Identifiler_v1	D21S11	-4.0	-4.0		-4.0	-4.0	-4.0	-4.0	NA		

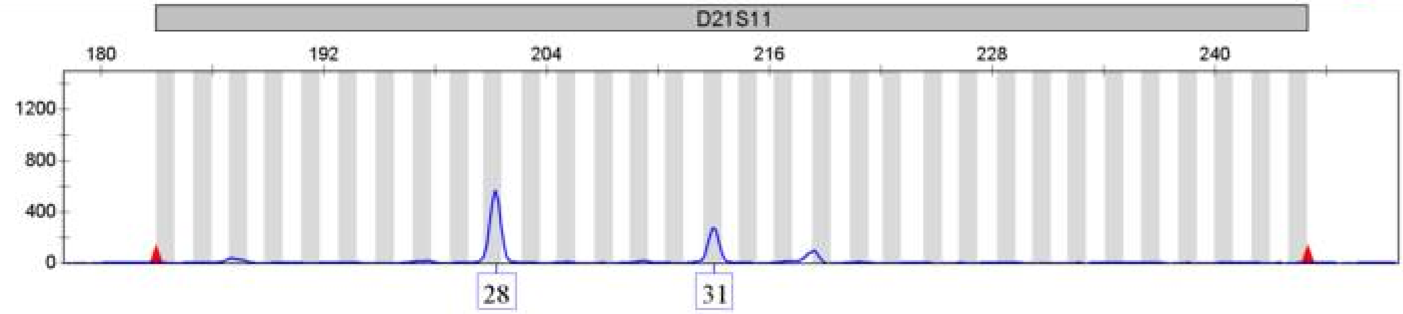

Sample Name	Panel	Marker	OS	BIN	PHR	LPH	SPU	AN	BD	CC	OVL	GQ
4660	Identifiler_v1	D2S1338	-4.0			-4.0	-4.0	-4.0	-4.0	NA		

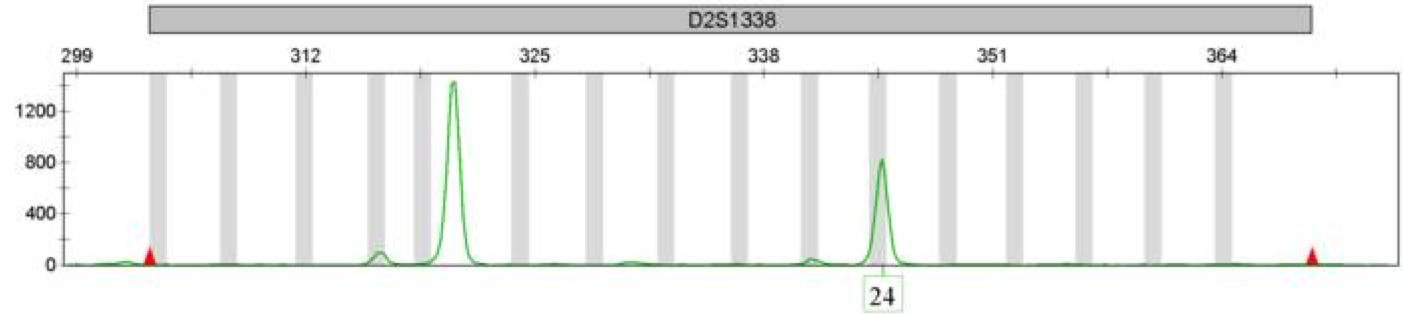

GeneMapper Software 5

Sample Name	Panel	Marker	OS	BIN	PHR	LPH	SPU	AN	BD	CC	OVL	GQ
4660	Identifiler_v1	D5S818	-4.0			-4.0	-4.0		-4.0	NA		
4660	Identifiler_v1	D8S1179	-4.0	-4.0		-4.0	-4.0	-4.0	-4.0	NA		
4660	Identifiler_v1	FGA	-4.0							NA		
4660	Identifiler_v1	TH01	-4.0	-4.0		-4.0	-4.0	-4.0	-4.0	NA		

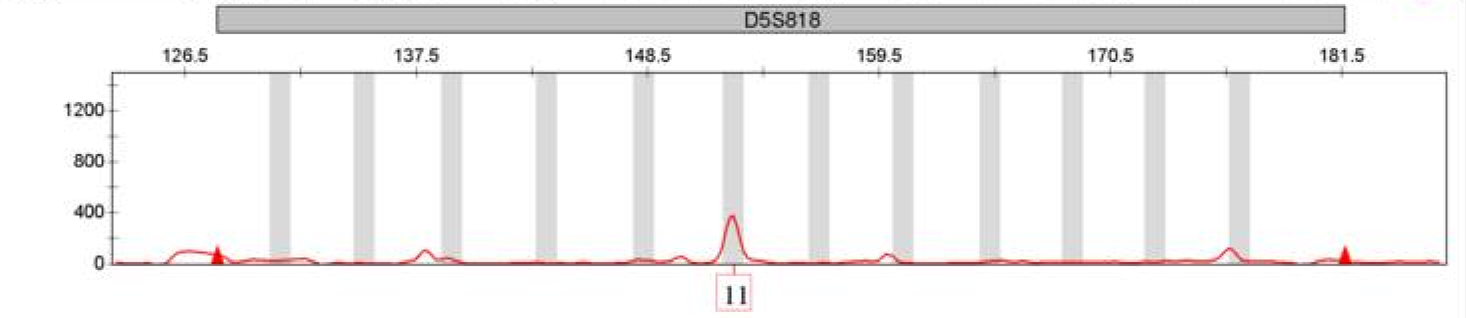

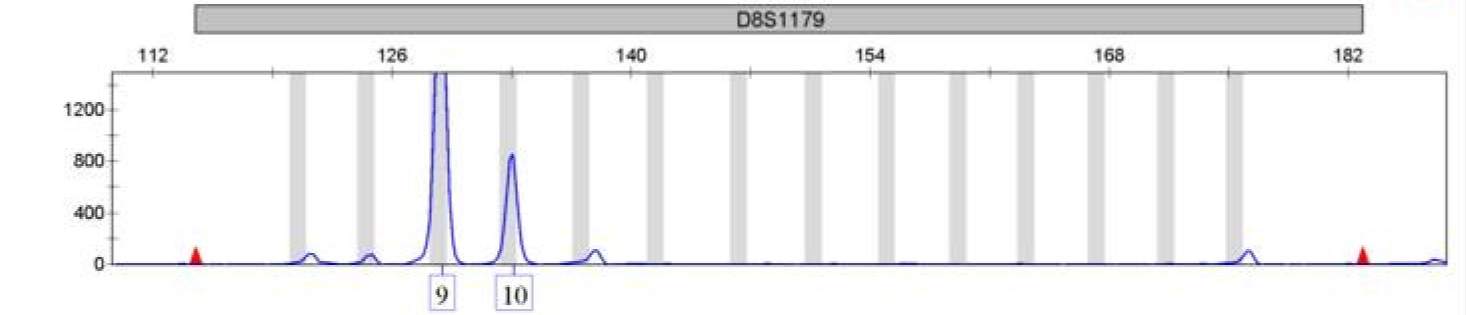

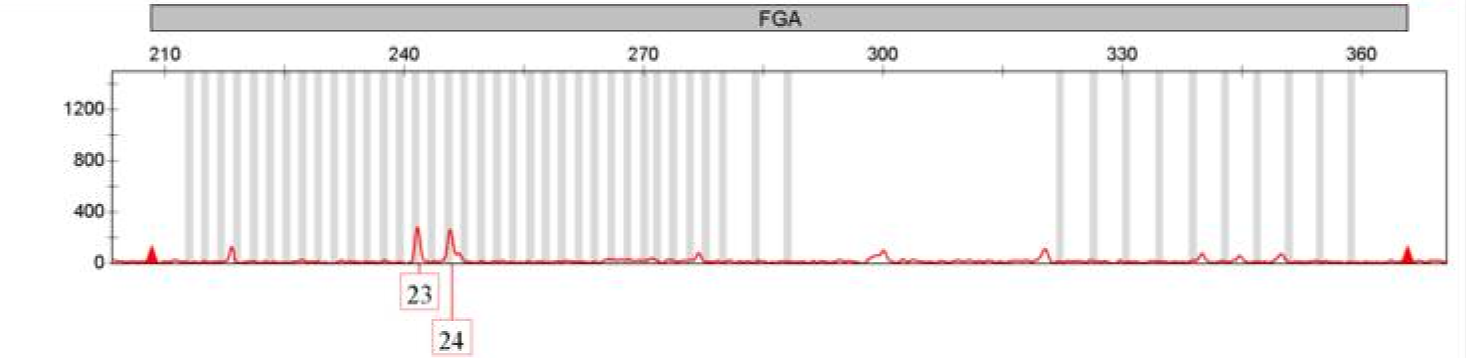

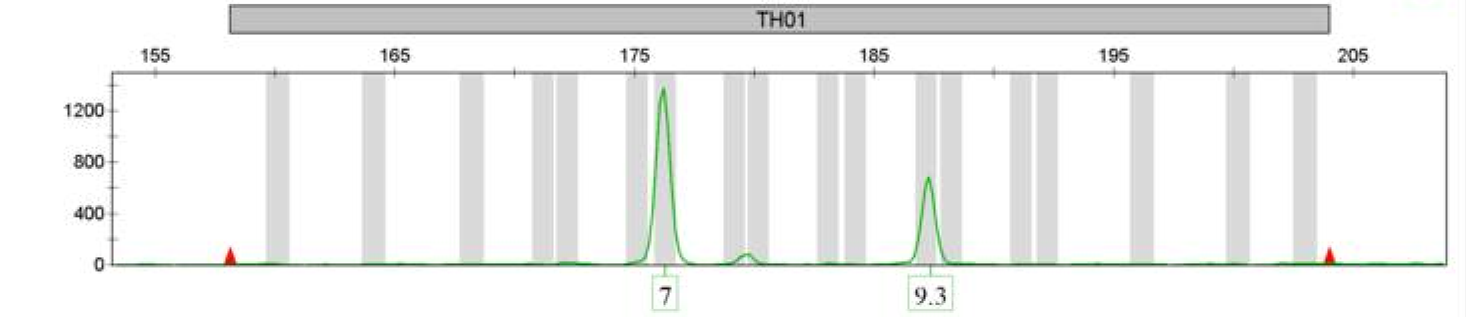

Supplement: Supplementary file 1 [file CAM4-7-3385-s001.pdf]
